# Relationship between evaluation of the teaching environment using DREEM scores and students’ school learning scores

**DOI:** 10.15694/mep.2019.000013.1

**Published:** 2019-01-14

**Authors:** Yukihiro Ikeda, Yoshie Kubota, Atsushi Hiraide

**Affiliations:** 1 Kindai University Hospital; 2 Faculty of Medicine; 3 Institutional Research Center

**Keywords:** medical school, students, DREEM, environment, learning

## Abstract

This article was migrated. The article was marked as recommended.

The results from a comprehensive survey of students’ perceptions of their educational environment using the Dundee Ready Educational Environment Measure (DREEM) in our Medical School were compared with students’ school learning scores. The subjects (n=495) were medical students beyond their first year of medical school. The students were asked to read each DREEM statement carefully and respond using a 5-point Likert-type scale, with responses ranging from strongly agree to strongly disagree. The mean total DREEM score was 113.4, and there was no significant difference among total DREEM scores for students in different school years. Sixth-year students scored significantly higher than those in the second year for the Academic Self-Perception and Social Self-Perception domains. Females had higher school learning scores and also had better total and Perception of Course Organizers DREEM scores. The DREEM score tended to be lower for those with lower school learning scores, with significant differences found for total, Academic Self-Perception and Social Self-Perception scores. This is the first study to use the DREEM score for Japanese medical students, and further prospective research is required to obtain a complete understanding of the results.

## Introduction

Evaluating and improving the learning environment of undergraduate students is an important task in medical education. The Dundee Ready Educational Environment Measure (DREEM) is a widely used and well-validated inventory for environmental evaluation by learners ( Al-Hazimi, Al-Hyiani and Roff, 2004; Al-Hazimi et al. 2004; Abraham et al. 2008; Bouhaimed, Thalib and Doi, 2009; Denz-Penhey and Murdoch 2009; Aghamolae and Fazel 2010; Bennett, Kelly and O’Flynn, 2010; Denz-Penhey and Murdoch 2010; Jakobsson, Danielsen and Edgren, 2011; Khan et al. 2011; Rotthoff et al. 2011; Hammond et al. 2012; Cocksedge and Taylor 2013; Dehghani et al. 2013; Al Faris et al. 2014; Al-Naggar et al. 2014; Pelzer, Hodgson and Were, 2014; Andalib et al. 2015; Bakhshialiabad, Bakhshi and Hassanshahi, 2015; Bhosale 2015; Kim et al. 2016; Mogre and Amalba 2016; Patil and Chaudhari, 2016; Chan et al. 2018). The review by Chan et al. (2018) included more than 100 studies conducted worldwide using the DREEM questionnaire. The DREEM is a 50-item, self-reported inventory that was designed by Roff et al. (1997) to measure the undergraduate medical educational environment. It comprises five subscales based on the student’s perception of teaching, perception of teachers, academic self-perception, perception of atmosphere, and social self-perception.

A practical guide ( McAleer and Roff, 2001) developed for interpretation of DREEM scores suggests that scores of 0-50 should be interpreted as “very poor,” 51-100 as “plenty of problems,” 101-150 as “more positive than negative,” and 151-200 as “excellent.” In the review by Chan et al. (2018), the mean total DREEM score over 98 studies was within the range of “more positive than negative” (101-150). Higher DREEM scores were associated with better past academic achievement, improved quality of life, higher resilience, positive attitudes toward the course, mindfulness, preparedness for practice, less psychological distress, and greater peer support. In a review by Soemantri et al., 12 learning environment instruments were identified for undergraduate medical education. Of these, the DREEM was found to be consistently reliable across different countries, cultures, and settings; thus, it was identified as the most suitable tool for measuring the educational environment in undergraduate medical education ( Soemantri, Herrera and Riquelme, 2010).

Although the DREEM has been used to examine the educational environment for students, the relationship between students’ learning scores and DREEM scores has not been widely investigated. Moreover, only one previous study has used the DREEM in Japan ( Tokuda et al. 2010) and none have determined DREEM scores for Japanese undergraduate students. Therefore, in this study, we conducted a survey of students’ perception of the educational environment using DREEM in our Medical School and analyzed the results in the context of the students’ school learning scores.

## Methods

### Subjects

The subjects were medical students who were beyond their first year. Students who agreed to participate in the survey on the first day of the new school year (in April in Japan) were enrolled in the study. We obtained approval for the study from the ethics committee of Kindai University faculty of Medicine.

### Data collection

The DREEM contains 50 statements related to a range of topics that are directly relevant to the educational environment ( Table 1). The questionnaire can be administered face-to-face in a classroom. Subjects were asked to read each statement carefully and respond on a 5-point Likert-type scale, with responses ranging from strongly agree to strongly disagree. DREEM questionnaires were completed by medical students in the second to sixth school year during orientation at the beginning of the year.

**Table 1.** DREEM score for each Item of study participants (n=495)

			mean	SD
1	Registrars’ Perception of Learning (PL)			
	1	I am encouraged to participate in teaching sessions	2.86	0.82
	7	The teaching is often stimulating	2.61	0.76
	13	The teaching is registrar centred	1.82	0.93
	16	The teaching helps to develop my competence	2.45	0.81
	20	The teaching is well focused	2.15	0.83
	22	The teaching helps to develop my confidence	2.07	0.82
	24	The teaching time is put to good use	2.21	0.80
	25	The teaching over emphasizes factual learning	1.79	0.73
	38	I am clear about the learning objectives of the course	2.20	0.70
	44	The teaching encourages me to be an active learner	2.23	0.81
	47	Long term learning is emphasized over short term learning	2.63	0.84
	48	The teaching is too teacher centred	1.91	0.72
2	Registrars’ Perception of Course Organisers (PC)			
	2	The course organisers are knowledgeable	2.96	0.70
	6	The course organisers espouse a patient centred approach to consulting	2.53	0.68
	8	The course organisers ridicule their registrars	2.41	0.87
	9	The course organisers are authoritarian	2.31	0.88
	18	The course organisers appear to have effective communication skills with patients	2.31	0.71
	29	The course organisers are good at providing feedback to registrars	2.00	0.73
	32	The course organisers provide constructive criticism here	2.28	0.71
	37	The course organisers give clear examples	2.34	0.72
	39	The course organisers get angry in teaching sessions	2.27	0.83
	40	The course organisers are well prepared for their teaching sessions	2.59	0.69
	50	The registrars irritate the course organisers	1.90	0.82
3	Registrars’ Academic Self-Perceptions(ASP)			
	5	Learning strategies which worked for me before continue to work for me now	2.48	0.86
	10	I am confident about passing this year	2.59	0.82
	21	I feel I am being well prepared for my profession	2.13	0.80
	26	Last year’s work has been a good preparation for this years work	2.50	0.75
	27	I am able to memorize all I need	1.62	0.87
	31	I have learned a lot about empathy in my profession	2.36	0.82
	41	My problem solving skills are being well developed here	2.19	0.73
	45	Much of what I have to learn seems relevant to a career in healthcare	2.84	0.73
4	Registrars’ Perceptions of Atmosphere (PA)			
	11	The atmosphere is relaxed during consultation teaching	2.32	0.71
	12	The course is well timetabled	1.63	0.99
	17	Cheating is a problem in this course	2.02	0.99
	23	The atmosphere is relaxed during lectures	2.46	0.68
	30	There are opportunities for me to develop interpersonal skills	2.31	0.83
	33	I feel comfortable in teaching sessions socially	2.50	0.81
	34	The atmosphere is relaxed during seminars/tutorials	2.54	0.70
	35	I find the experience disappointing	2.09	0.91
	36	I am able to concentrate well	2.16	0.83
	42	The enjoyment outweighs the stress of studying medicine	1.97	0.83
	43	The atmosphere motivates me as a learner	2.07	0.82
	49	I feel able to ask the questions I want	2.17	0.80
5	Registrars’ Social Self-Perceptions(SSP)			
	3	There is a good support system for registrars who get stressed	1.68	0.93
	4	I am too tired to enjoy this course	1.84	0.85
	14	I am rarely bored on this course	1.60	0.88
	15	I have good friends in this course	3.11	0.71
	19	My social life is good	2.46	0.77
	28	I seldom feel lonely	2.37	0.88
	46	My accommodation is pleasant	2.88	0.76

### Data preparation

Items were scored as follows: 4 strongly agree (SA), 3 agree (A), 2 uncertain (U), 1 disagree (D), and 0 strongly disagree (SD). However, 9 of the 50 items (numbers 4, 8, 9, 17, 25, 35, 39, 48, and 50) are negative statements and were scored as 0 for SA, 1 for A, 2 for U, 3 for D, and 4 for SD. The maximum score for the 50-item DREEM was 200, indicating an ideal educational environment as perceived by the subject. The interpretation of the overall score ( McAleer and Roff 2001) is 0-50 very poor, 51-100 plenty of problems, 101-150 more positive than negative, and 151-200 excellent. A score of 100 indicates an environment perceived to be ambivalent by the students and one that requires improvement. The DREEM can also be used to pinpoint more specific strengths and weaknesses within the educational climate. To achieve this, the individual responses to items need to be assessed. Items with a mean score of ≥3.5 are positive points. Any item with a mean score ≤2 requires close examination as this may indicate a problem area. Items with a mean score between 2 and 3 indicate educational areas that could be improved ( McAleer and Roff 2001). The obtained data were digitized and combined with the rank in school learning scores, after which the data were anonymized.

### Statistical analyses

Statistical analyses were conducted using SPSS (Statistical Package for Social Sciences) ver. 24 (IBM Corp.). Analysis of variance was performed for comparisons with school years and gender; if the results were significant, pairwise comparisons were then performed. Scheffé multiple comparison was used to adjust the level of significance to 5% when five groups were compared. The relationship between the rank in school learning scores and DREEM scores was analyzed using a Mann-Whitney U-test. In all cases, p < 0.05 was considered to be significant.

## Results/Analysis

The survey participation rate was high, at 82.8% (495/598). Each item was classified into five categories of subjects’ Perception of Learning (PL), Perception of Course Organizers (PC), Academic Self Perception (ASP), Perceptions of Atmosphere (PA), and Social Self Perception (SSP) ( McAleer and Roff 2001). Items with a score of <2 points were “Irritated by course organizers,” “I am able to memorize all I need,” “The course is well timetabled,” “Enjoyment outweighs the stress of studying medicine,” “There is a good support system for students who become stressed,” “I am too tired to enjoy this course,” and “I am rarely bored in this course.”

**Figure 1.**
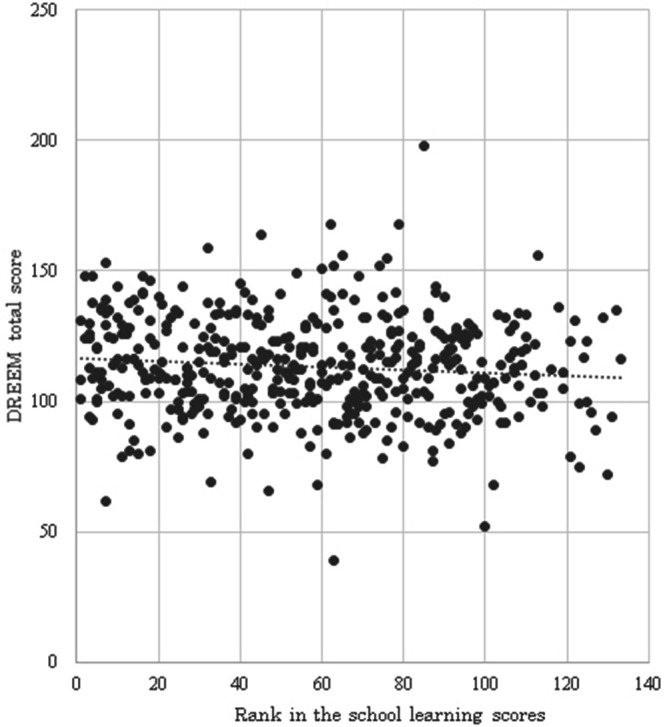
Scatter plot of DREEM total scores and rank in school learning scores

Total DREEM scores and scores for the five categories for all subjects are shown in Table 2. There was no significant difference in total DREEM scores among students in different school years. Students in the sixth year scored significantly higher than those in the second year for ASP and SSP ( Table 2). Females had a higher rank in school learning scores ( Table 3) and also had better Total and PC DREEM scores ( Table 3). The DREEM score tended to be lower for students with a lower rank in school learning scores ( Figure 1), with significant differences for Total, ASP and SSP scores ( Table 4).

**Table 2.** Total score and subscales of the DREEM by school years

School years	2 (n=116)			3 (n=85)			4 (n=94)			5 (n=108)			6 (n=92)			Total (n=495)		
Total	112.8	±	18.4	114.7	±	21.0	112.2	±	19.6	110.5	±	17.4	117.3	±	17.5	113.4	±	18.8
Perception of Learning	27.0	±	5.2	27.1	±	5.9	26.8	±	5.8	26.0	±	5.3	27.5	±	4.8	26.9	±	5.4
Perception of Course organisers	25.2	±	4.1	26.1	±	4.9	26.4	±	5.1	25.3	±	4.4	26.5	±	4.5	25.8	±	4.6
Academic Self-Perception	18.0	±	4.0 ^a^	19.0	±	4.2 ^ab^	18.9	±	3.3 ^ab^	18.2	±	3.3 ^ab^	19.8	±	3.7 ^b^	18.7	±	3.7
Perceptions of Atomosphere	26.5	±	5.4	26.6	±	5.7	25.1	±	5.8	25.6	±	4.7	27.1	±	5.4	26.2	±	5.4
Social Self Perceptions	16.0	±	2.9 ^a^	15.8	±	3.5 ^ab^	15.6	±	3.4 ^ab^	15.5	±	2.8 ^ab^	16.8	±	2.7 ^b^	15.9	±	3.1

Values are means ± SD. Within a subscale group, means in a line with superscripts without a common letter differ, p<0.05

**Table 3.** Total score, subscales of the DREEM and rank in the school learning score by gender

	Men (n=323)			Women (n=172)		
Total ^*^	112.1	±	19.0	115.7	±	18.2
Perception of Learning	26.6	±	5.6	27.4	±	5.1
Perception of Course organisers ^**^	25.3	±	4.6	26.8	±	4.4
Academic Self-Perception	18.6	±	3.9	18.9	±	3.4
Perceptions of Atomosphere	26.1	±	5.4	26.4	±	5.5
Social Self Perceptions	15.8	±	3.1	16.3	±	3.1
Rank in the school learning score ^1^	60.9	±	34.6	51.2	±	32.4

Values are means ± SD.

^**^ p<0.01.

^*^ p<0.05 statistical significant by ANOVA.

^1^ p<0.01 statistical significant by Mann-Whitney U-test.

**Table 4.** Spearman correlation coefficients for rank in school learning scores and DREEM subscales

	Rank in the school learning scores
Total ^*^	-0.099
Perception of Learning	-0.066
Perception of Course organisers	-0.051
Academic Self-Perception ^***^	-0.222
Perceptions of Atomosphere	-0.086
Social Self Perceptions ^*^	-0.113

P-values of correlation of coefficients are

^***^ p<0.001.

^*^ p<0.05.

## Discussion

In this study, we examined the relationships among school years, rank in school learning scores, gender, and DREEM scores in Japanese medical school students. The mean total DREEM score was 113.4, which is comparable to those reported in medical schools worldwide (Al-Hazimi, Al-Hyiani, and Roff, 2004; Al-Hazimi et al. 2004; Abraham et al. 2008; Bouhaimed, Thalib and Doi, 2009; Denz-Penhey and Murdoch 2009; Aghamolae and Fazel 2010; Bennett, Kelly and O’Flynn, 2010; Denz-Penhey and Murdoch 2010; Jakobsson, Danielsen and Edgren, 2011; Khan et al. 2011; Rotthoff et al. 2011; Hammond et al. 2012; Cocksedge and Taylor 2013; Dehghani et al. 2013; Al Faris et al. 2014; Al-Naggar et al. 2014; Pelzer, Hodgson and Were, 2014; Andalib et al. 2015; Bakhshialiabad, Bakhshi and Hassanshahi, 2015; Bhosale 2015; Kim et al. 2016; Mogre and Amalba 2016; Patil and Chaudhari, 2016; Chan et al. 2018). The score suggests that the undergraduate medical education program at Kindai University has more positive aspects than negative, according to the interpretation recommended in the DREEM practical guide ( McAleer and Roff 2001).

Total DREEM scores did not differ significantly among students from different school years, as also found in previous studies ( Avalos, Freeman and Dunne, 2007; Al-Mohaimeed 2013). However, other studies have found that DREEM scores are high in earlier school years ( Pai et al. 2014; Palés et al. 2015; Enns et al. 2015). In the current study, fifth year students showed a tendency for a lower total DREEM score compared to students in other years, but the difference was not significant. Similar results have been described in previous studies ( Demirören et al. 2008; Enns et al. 2015; Xu et al. 2016). In Japan, medical students commonly study mainly in a lecture style up to the middle of the fourth year, and it is generally a big step to enter clinical training for fifth-year students. Stress may be exacerbated in the training years, and students in this phase reported that they really notice or feel the lack of a good support program, are too tired to enjoy their studies, and have difficulties in their school life. Students in clinical training also perceived that the teaching was not cohesive, focused, stimulating, or student-centered, and did not develop their confidence. These students also indicated that their teachers do not give appropriate feedback, which we find especially worrying in a clinical setting that demands frequent feedback. Therefore, clinical training may be the reason why the total score for advanced students is low in some studies.

Lower scores in the clinical training years could also be explained by the greater workload and more responsibilities given to students in this phase. In addition, clinical work during training may produce more stress in trainees and faculty, which may contribute to moral crises and emotional abuse. However, we believe that the scores for items such as ASP and SSP will rise with progression in medical education and age. Our results showed that there was no difference among school years for total DREEM scores. However, ASP and SSP scores were higher for students in their sixth year, and these scores for sixth-year students were significantly higher than those for second-year students. At our university, clinical training is completed and the program for the national exam is started in the sixth year, and these changes may be factors that raise DREEM scores for sixth-year students.

Several reports have shown no gender-based effects in DREEM scores ( Carmody et al. 2009; Edgren et al. 2010; Al-Mohaimeed et al. 2013; Karim et al. 2015; Condon et al. 2017), but others have shown higher scores for females ( Bassaw et al. 2003; Jawaid et al. 2013; Belayachi et al. 2015; Rahman et al. 2015; Rehman et al. 2016). We found only one study that reported higher scores in males ( Finn, Avalos and Dunne, 2014). In the current study, we observed higher DREEM total and PC scores for females. One of the reasons for this may be that females had a higher rank in school learning scores ( Table 3, p<0.01), and female gender may be a surrogate variable for a higher rank in school learning scores, since our results showed that a higher rank was related to a higher DREEM score ( Figure 1). However, this was a retrospective study; therefore, it is unclear whether the DREEM score affects the rank in learning scores. The learning environment changes according to the perception of the person him/herself; therefore, an increase in DREEM score may improve the rank in school learning scores. Further research is warranted to examine this hypothesis. The high PC score for females is consistent with female students in Japan evaluating teachers with higher scores. These data are not shown, but females tended to give higher scores for teachers in other classroom evaluations conducted by students.

Items with scores <2 indicate weak points in our program that need improvement. The scores for “I am able to memorize all I need,” “The course is well timetabled,” “There is a good support system for students who become stressed,” and “I am too tired to enjoy this course” were particularly low. Weaknesses were also frequently observed in the SSP, which shows that social support for students is necessary. To our knowledge, this is the first study to use the DREEM score for Japanese medical students. The participation rate exceeded 80%, indicating the success of the survey. However, the study has a limitation of being a cross-sectional survey conducted in a single medical school, and the results should be extrapolated and interpreted with caution. To overcome this limitation, it is necessary to extend the investigation to more medical universities in Japan. Further prospective research is also needed to obtain a complete understanding of the relationship of DREEM scores with student learning.

## Conclusion

This is the first study to use the DREEM score for Japanese medical students, and further prospective research is required to obtain a complete understanding of the results.

## Take Home Messages

• Students’ perceptions of their educational environment using DREEM scores were compared with their school learning scores.
• The mean total DREEM score was 113.4, with no significant difference among total DREEM scores for students in different school years.
• Sixth-year students scored significantly higher than second-year students for the DREEM domains of Academic Self-Perception and Social Self-Perception.
• Females had higher school learning scores and also had higher total DREEM scores.
• DREEM scores tended to be lower for students with lower school learning scores.

## Notes On Contributors

Dr. Yukihiro Ikeda, Lecturer, Kindai University Hospital. ORCID: https://orcid.org/0000-0002-2768-4145

Dr. Yoshie Kubota, Assistant Professor, Faculty of medicine, Kindai University.

Prof. Atsushi Hiraide, Instututional Research Center, Kindai University. ORCID: https://orcid.org/0000-0001-8999-0562
