# Supplementary material for: Relationship between evaluation of the teaching environment using DREEM scores and students’ school learning scores
Source: MedEdPublish (2016). 2019 Jan 14;8:13. [Version 1] doi: 10.15694/mep.2019.000013.1 (PMC10712524; doi:10.15694/mep.2019.000013.1)
Supplement: Peer review history

# Reviewer report: Reviewer response for version 1

**DOI:** 10.21956/mep.19744.r29605

**Published:** 2019-01-27

**Authors:** Peter Dieter

**Affiliations:** 1 AMSE

This review has been migrated. The reviewer awarded 3 stars out of 5 The authors showed the results of a survey of students´ perceptions of their educational environment using DREEM and compared the results with students learning scores. The students were from years 2-6. The study was well performed and showed almost no differences of students from different years, slight better results for female students and a correlation between DREEM and students learning scores. The mean total DREEM score was 113.4 (“more positive than negative”). A similar survey should be performed at other medical schools in Japan.

Reviewer Expertise:

NA

No decision status is available

# Reviewer report: Reviewer response for version 1

**DOI:** 10.21956/mep.19744.r29604

**Published:** 2019-01-15

**Authors:** Balakrishnan Nair

**Affiliations:** 1 Centre for Medical Professional Development and University of Newcastle

This review has been migrated. The reviewer awarded 4 stars out of 5 Evaluation of undergraduate learning environment is important . In this paper , the authors used the well known and validated DREEM score to evaluate this in Japan . This is the first study using this measure in Japan . The sample size is very adequate and the response rate was 84%; both are excellent . The study concluded that the learning environment is satisfactory from the student point of view . The mean was in the “ more positive than negative “range . This is a well done and written study. I hope other schools will emulate this. The next question is how do we improve the environment to “excellent”; it will be good to see what students think should be done by us to achieve that . I look forward to the follow up paper

Reviewer Expertise:

NA

No decision status is available
